# Memory T Cell Subsets Expressing Tissue Homing Receptors and Chemokine Levels in Human Tegumentary Leishmaniasis

**DOI:** 10.3390/cells14080604

**Published:** 2025-04-16

**Authors:** Julia Pimentel, M. Fernanda García Bustos, Paula Ragone, Jorge D. Marco, Paola Barroso, Andrea Cecilia Mesías, Mercedes Basombrío, María Occhionero, Federico Ramos, Susana Adriana Laucella, Cecilia Pérez Brandán, Cecilia Parodi

**Affiliations:** 1 Instituto de Patología Experimental, CONICET/Universidad Nacional de Salta, Salta A4408FVY, Argentina; juliapimen04@gmail.com (J.P.); morfofisiounsa@gmail.com (M.F.G.B.); p_ragone@yahoo.com.ar (P.R.); diegomarcoar@gmail.com (J.D.M.); barrosopaola75@gmail.com (P.B.); andreacmesias@gmail.com (A.C.M.); ipe.unsa@gmail.com (M.B.); chelymao94@gmail.com (M.O.); federamosqui@gmail.com (F.R.); perezbrandan@gmail.com (C.P.B.); 2 Instituto Nacional de Parasitología Dr. Mario Fatala Chaben, Departamento de Investigación, Buenos Aires C1282AFF, Argentina; slaucella@yahoo.com

**Keywords:** tegumentary leishmaniasis, cutaneous leishmaniasis, mucosal leishmaniasis, chemokine receptors, memory T lymphocytes, chemokines, CCR4, CCR6, CCR3, CCR10, CCL17, CCL20

## Abstract

Tegumentary leishmaniasis (TL) presents two main clinical forms: cutaneous (CL) and mucosal (ML) leishmaniasis affecting skin and nasopharyngeal mucosa. Due to parasite localization through disease stages, recruitment of T cells expressing chemokine receptors and their ligands will influence the generated host responses. The aim of this work was to characterize differential profiles of T cells expressing chemokine receptors and their plasma ligands by flow cytometry and ELISA. CL patients showed increased numbers of effector memory CD4^+^ T cells expressing skin homing receptors (CLA, CCR4), with the reversion of this effector phenotype observed after achieving clinical recovery. Meanwhile, ML patients showed higher frequencies of effector memory/terminal effector CD4^+^ and CD8^+^ T cells expressing chemokine receptors directed to skin (CLA, CCR4, CCR10) and mucosal (CCR6) tissues. Additionally, we reported that plasma amounts of ligands (CCL17, CCL20) vary according to the clinical form of TL. Finally, we demonstrated the ability of *Leishmania* spp. to modulate chemokine production (CCL17) in vitro. This work highlights the effector T cell response directed to skin and mucosal tissues in TL, emphasizing the role of cytotoxic functions in ML. The studied chemokine receptors could contribute to predicting disease progression and guiding future studies targeting relevant receptors to diminish pathogenic effector functions.

## 1. Introduction

Tegumentary leishmaniasis (TL) is a disease strongly linked to poverty and unhealthy living conditions with a worldwide incidence and nearly 700,000 to 1,000,000 new cases reported per year (WHO report 2023). The etiological agent is an intracellular parasite from the genus *Leishmania,* and the disease begins with the bite of an infected phlebotomine of the genus *Lutzomyia* in the Americas [1]. TL is endemic in Latin America [2] and presents two main clinical forms affecting skin or nasopharyngeal mucosa. The presence of skin ulcers with delimited bounds [3] is a main feature of cutaneous leishmaniasis (CL). Months or years after the onset of primary cutaneous lesions, mucosal leishmaniasis (ML) can appear, compromising oral and nasopharyngeal tissues. These mucosal lesions may provoke facial disfiguration and distress to breath, adding the social burden associated to these types of injuries [1,2,4,5]. In the northwest of Argentina, the most common causative agent is *L. (Viannia) braziliensis* followed by *L. (Leishmania) amazonensis* [6]. The mounted immune response to *Leishmania* spp. infection starts from the first encounter of the parasite with neutrophils which fail in neutralizing the infecting protozoan, acting as Trojan horses and becoming parasite hosts [5]. Then, macrophages and dendritic cells arrive to phagocyte parasites, leading to clearance or acting as vehicles allowing for the dissemination of the pathogen [1]. While CD4^+^ T cells are critical for the control of *Leishmania* spp. infections, CD8^+^ T cells play a controversial role [7]. However, both T cell subsets are implicated in TL outcomes [8]. Moreover, chemokines orchestrate the migration of cell types carrying tissue homing receptors to inflamed tissues. TL involves the skin and nasopharyngeal mucosa, suggesting that the generated chemokine signals and the recruited leukocyte subsets will vary depending on the preferential location of the parasite. The implication of memory T cell subpopulations harboring specific homing receptors and their ligands in human TL has not been fully elucidated [9,10], especially when referring to the mucosal clinical form [11]. Increased expression of skin homing receptors as cutaneous lymphocyte-associated antigen (CLA) [10] and CCR4 [8] was found by analyzing cutaneous lesions of CL patients. However, evaluation of the expression of CCR6 in relation to protection or severity during mice infections with *L. major* [12,13] and CCR3 in cutaneous lesions [14] led to inconclusive findings.

In the present study, we characterized the peripheral memory T cell subsets expressing a panel of chemokine receptors directed to skin and mucosal tissues, the plasma amounts of their ligands, and the association of these parameters with the clinical form, disease duration, and outcomes in TL. In relation to the role of the parasite, we demonstrated the ability of *Leishmania* spp. to modulate the production of chemokines by using in vitro assays. This work intends to contribute to improving the patients’ clinical follow up and to be useful in future experimental approaches targeting specific cellular receptors.

## 2. Materials and Methods

### 2.1. Study Groups

The study population included patients aged between 12 and 86 years, residing in Salta province—an endemic area for tegumentary leishmaniasis (TL) located in the northwest of Argentina. The sample size (*n*) of active TL patients (*n* = 56) was calculated using the statistical formula for estimating proportions in finite populations [15,16], where *p* is the estimated proportion of the population with the desired characteristic, and *q* = 1 − *p*. The calculation was performed with a margin of error (*e*) of 10%, a confidence level (*z*) of 99%, and a population size (*N*) of 130, corresponding to the average number of TL patients diagnosed annually in Salta province over the last 10 years. The formula used for the sample size calculation is as follows [15,16]:

*n* = (z² × *p* × q × N)/[e² × (N − 1) + z² × *p* × q]

TL diagnosis was performed on the basis of clinical evaluation, the detection of amastigotes in Giemsa-stained lesion-scraping smears, parasite isolation from lesions and cultures in blood agar, and *Leishmania* DNA detection by using k-PCR [kinetoplast DNA (kDNA) PCR] [17]. Patients with comorbidities such as diabetes or HIV were excluded. Study groups were classified as follows: cutaneous leishmaniasis patients (CL, *n* = 32) presenting single, two, or several cutaneous lesions; CL recovered patients with healed lesions (CL-R, *n* = 13); and mucosal leishmaniasis patients (ML, *n* = 24) presenting mucosal nasopharyngeal lesions. Healthy subjects (HS, *n* = 17) with no leishmaniasis history were chosen as the control group. Demographic and clinical data are shown in Table 1. The analytical approaches were performed independently of the condition for anti-*T cruzi* antibodies after verifying that there were no significant differences between CL vs. *T. cruzi*-seropositive CL and ML vs. *T. cruzi*-seropositive ML groups.

### 2.2. Ethics Statement

The present study was approved by the Provincial Commission for Biomedical Research, Secretariat for Organizational Development, Ministry of Public Health of Salta (Exp. N° 321-136934/2018-0, approval date 19 June 2019). All subjects gave written informed consent in accordance with the 1964 Declaration of Helsinki.

### 2.3. Blood Sampling Procedure

Peripheral blood samples (20 mL) were obtained from TL patients and healthy subjects. Plasma samples were obtained by centrifugation at 400× *g* for 10 min and stored at −80 °C. Peripheral blood mononuclear cells (PBMCs) were isolated following the Ficoll–Paque TM Plus density gradient separation method by centrifugation at 400× *g* for 40 min. Cells were washed with PBS 1x. Next, an aliquot of fresh PBMCs was used to perform cell culture assays, and the rest of the isolated cells were cryopreserved at −140 °C in aliquots of 5 × 10^6^ cells/mL using a solution of FBS (Fetal Bovine Serum, Gibco™, New York, NY, USA) and 10% DMSO (Dimethyl Sulfoxide, Sigma-Aldrich, Burlington, MA, USA) for later flow cytometry determinations.

### 2.4. Anti-T. Cruzi Antibody Analysis

To discriminate among TL patients that were also reactive to *T. cruzi*, anti-*T. cruzi* antibodies have been tested using a commercial enzyme-linked immunosorbent assay (ELISA) based on the use of recombinant *T. cruzi*-derived proteins as antigens (recombinant ELISA v.4.0, Wiener, Rosario, Argentina) following the supplier’s instructions.

### 2.5. Chemokine Receptor Determination

Expression of chemokine receptors was assessed by flow cytometry. PBMCs from TL patients cryopreserved at −140 °C were thawed, viability was measured using Trypan-blue dye exclusion, and then the cells were labeled with anti-human monoclonal antibodies as follows: FITC anti-CD45RO; PE anti-CLA; PE anti-CCR10; PeCy7 anti-CD27; PerCPCy5.5 anti-CCR6; PerCPCy5.5 anti-CCR3; APC anti-CCR4; APCH7 anti-CD4; and APCH7 anti-CD8 (BD Pharmingen TM, New York, NY, USA). For ensuring homing receptor stability on thawed PBMCs, we performed duplicate determinations in chosen TL and HS samples. Data acquisition and analysis were executed using FACs Diva Software 7 in a BD FACSCanto II cytometer.

### 2.6. Soluble Leishmania Antigens (SLAs)

*Leishmania* lysates composed of promastigotes derived from the predominant *Leishmania* spp. found in the northwest of Argentina, *L. (V.) braziliensis* (MHOM/BR/75/M2903) and *L. (L.) amazonensis* (MHOM/VE/84/MEL), were obtained. Massive cultures of promastigotes in the exponential phase of growth of both strains were separately processed. Briefly, a first centrifugation step (1500 rpm, 10 min) was performed to remove culture media remnants and parasite pellets were then suspended on PBS 1x followed by a second centrifugation step (1500 rpm, 20 min, 4 °C). Then, a volume of 1.2 mL of lysis buffer (100 mM Tris-HCl, 1mM EDTA, pH = 8) was added to 10^8^ parasites. After a short incubation period of 10 min at 4 °C, three cycles of freezing/thawing in liquid nitrogen containers and in a 37 °C water bath were performed. Then, parasite samples were subjected to sonication (3 pulse cycles of 1 min; 40 watts; 80% amplitude) and complete lysis of the parasite was verified. Finally, a centrifugation step (20,800 rpm, 20 min, 4 °C) with posterior filtration (0.45 μm pore size) of the collected supernatant was performed to obtain the sterile SLAs. Supernatant aliquots were stored at −80 °C until use. Protein content was measured by using the Bradford method.

### 2.7. In Vitro PBMC Cultures

Fresh isolated PBMCs were cultured (1 × 10^6^ cells/mL) in 24-well plates in the presence or absence of soluble *Leishmania* antigens composed of equal concentrations (20 μg/ mL) of *L. (V.) braziliensis* and *L. (L.) amazonensis*. PBMC cultures were performed over 7 days at 37 °C in a 5% CO_2_ atmosphere incubator. After that time, supernatants were collected and stored at −80 °C for later determination.

### 2.8. Chemokine and Cytokine Quantification

To determine in vitro production and peripheral concentrations of chemokines, the levels of CCL17 (pg/mL), CCL20 (pg/ mL), IFN-γ, and IL-10 were assessed in culture supernatants and plasma samples by using commercial enzyme-linked immunosorbent assay (ELISA) kits. The assays were carried out following the manufacturer’s protocol (DuoSet^®^ ELISA for CCL17 and CCL20 determinations, as well as IFN-γ and IL-10 measurements using R&D Systems, Minneapolis, USA and BD OptEIATM, New York, USA).

### 2.9. Statistical Analysis

A level of *p* < 0.05 was accepted as being statistically significant. The normality of the variable distribution was assessed by the D’Agostino & Pearson omnibus test. Continuous variables between two groups were compared with the Mann–Whitney U test. A nonparametric Kruskal–Wallis test with Dunn’s post-test and trend analyses with an ANOVA test were used to compare differences among three or more groups. A two-way ANOVA and Bonferroni’s post-test were used to investigate the effects of two different independent variables and verify if there is a significant main effect of each variable. The data were analyzed with GraphPad Prism 9.0.2 Software, California, USA.

## 3. Results

### 3.1. Peripheral CLA, CCR4, CCR6, CCR3, and CCR10 Expression on Total CD4^+^ and CD8^+^ T Cells in TL Patients

Circulating skin homing populations are defined by the expression of CLA along with receptors such as CCR4, CCR6, and CCR10, whereas CCR3 and CCR6 are also involved in trafficking of T cells to mucosal tissues. Patients with ML showed increased percentages of total CD4^+^ T cells expressing CLA compared to healthy subjects (Table 2). ML patients also showed higher numbers of total CD4^+^ and CD8^+^ T cells expressing CCR3 in comparison with healthy subjects and CL patients. In addition, the ML group showed increased values of total CD4^+^ and CD8^+^ T cells expressing CCR6 compared with CL subjects and a positive trend toward higher expression of CCR4^+^CD4^+^ T cells and CCR10^+^CD8^+^ T cells in comparison with CL and healthy controls (Table 2).

### 3.2. Memory T Cell Populations Expressing Tissue Tropism Receptors

**Figure 1:**
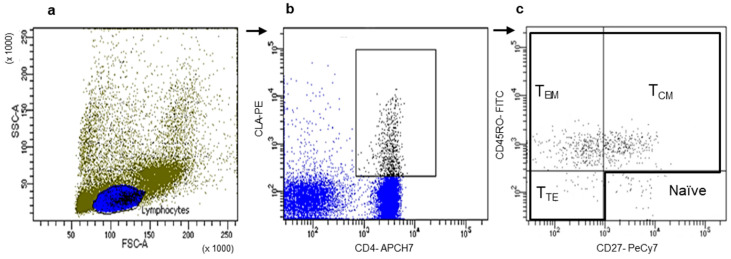
Representative gating strategy of memory T cell subsets expressing chemokines receptors. (**a**) Lymphocytes (blue) were initially gated by forward and side light scatter, (**b**) CLA^+^CD4^+^ co-expression was selected (dark gray), and (**c**) a dot plot graph of CD45RO and CD27 staining of the gated CLA^+^CD4^+^ T cells are shown. Memory T cell populations were determined as central memory (T_CM_, CD45RO^+^CD27^+^), effector memory (T_EM_, CD45RO^+^ CD27^—^), and terminal effector (T_TE_, CD45RO^—^CD27^—^) cells.

We selected CD4^+^ or CD8^+^ T cells expressing each chemokine receptor and then evaluated the frequencies of the three main memory T cell populations, T_CM_ (central memory), T_EM_ (effector memory), and T_TE_ (terminal effector), according to the expression of CD45RO and CD27 on both T cell subsets (Figure 1).

**Figure 2:**
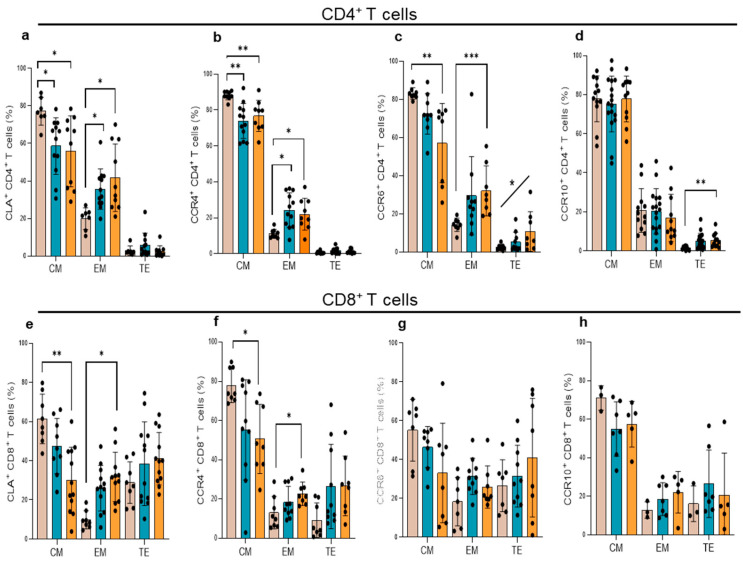
CLA, CCR4, CCR6, and CCR10 memory T cell populations in TL patients. (**a**–**d**) CD4^+^ T cells and (**e**–**h**) CD8^+^ T cells from (HS, gray bars) healthy subjects, (CL, blue bars) cutaneous leishmaniasis patients, and (ML, orange bars) mucosal leishmaniasis (ML) patients. Memory T cell populations were determined as central memory (T_CM_, CD45RO^+^CD27^+^), effector memory (T_EM_, CD45RO^+^CD27^—^), and terminal effector (T_TE_, CD45RO^—^CD27^—^) cells. Significant differences between memory T cell subsets were recorded using the Kruskal–Wallis test and Dunn’s post-test. (*) *p* < 0.05; (**) *p* < 0.01, (***) *p* < 0.001 The asterisk over the diagonal line indicates a positive linear trend using a one-way ANOVA with a post-test for linear trends (*p* < 0.05).

A similar profile was observed for CLA and CCR4 expression in CL and ML patients, with diminished frequencies of T_CM_ and increased numbers of T_EM_ CD4^+^ T cells (Figure 2a,b). On CD8^+^ T cells, the percentages of T_CM_ CD8^+^ T cells expressing these chemokine receptors significantly decreased and those of T_EM_ increased only in ML patients (Figure 2e,f). In contrast, no alterations of T_CM_, T_EM_, and T_TE_ CD8^+^ T cells expressing these chemokine receptors were observed in CL patients. In addition, ML patients showed diminished percentages of T_CM_, increased frequencies of T_EM_, and a positive trend toward higher numbers of T_TE_ CD4^+^ T cells expressing CCR6 (Figure 2c). Higher percentages of T_TE_ CD4^+^CCR10^+^ T cells were also recorded in ML patients (Figure 2d). No differences in CCR6^+^ and CCR10^+^ memory T cell populations were recorded in CL patients (Figure 2c,d,g,h), and CCR3 expression did not vary between CL and ML in the different memory T cell subsets analyzed.

**Figure 3:**
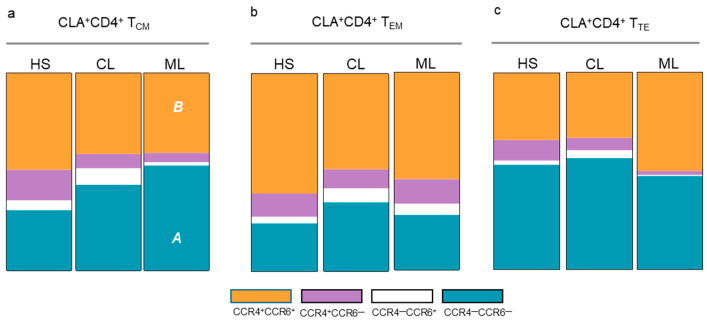
Co-expression of CCR4 and CCR6 on CLA^+^CD4^+^ T_CM_, CLA^+^CD4^+^ T_EM_, and CLA^+^CD4^+^ T_TE_ fractions in TL patients. CCR4^+^CCR6^+^ (orange), CCR4^+^CCR6^—^ (violet), CCR4^—^CCR6^+^ (white), and CCR4^−^CCR6^−^ (blue) fractions of (**a**) CLA^+^CD4^+^ T_CM_ cells and (**b**) CLA^+^CD4^+^ T_EM_ cells and (**c**) CLA^+^CD4^+^ T_TE_ cells from (HS) healthy subjects, (CL) cutaneous leishmaniasis patients, and (ML) mucosal leishmaniasis patients. (*A*) CCR4^−^CCR6^−^CLA^+^, *p* < 0.05, ML vs. HS; (*B*) *p* < 0.01, CCR4^+^CCR6^+^CLA^+^, ML vs. HS. Significant differences were assessed using the Kruskal–Wallis test and Dunn’s post-test.

Next, co-expression profiles of chemokine receptors were assessed to identify CD4^+^ T cell subsets with homing characteristics (Th1: CCR4^—^CCR6^—^CLA^+^; Th22 and Th17: CCR4^+^CCR6^+^CLA^+^). ML patients showed a significant decrease in T_CM_ CCR4^+^CCR6^+^CLA^+^CD4^+^ T cells and increased numbers of T_CM_ CCR4^—^CCR6^—^CLA^+^CD4^+^ T cells compared to healthy subjects (Figure 3a). In contrast, no differences in T_EM_ CLA^+^CD4^+^ and T_TE_ CLA^+^CD4^+^ T cells were observed between CL patients, LM patients, and healthy subjects (Figure 3b,c).

### 3.3. Memory T Cell Subsets in CL Patients Recovered from Their Cutaneous Lesions

**Figure 4:**
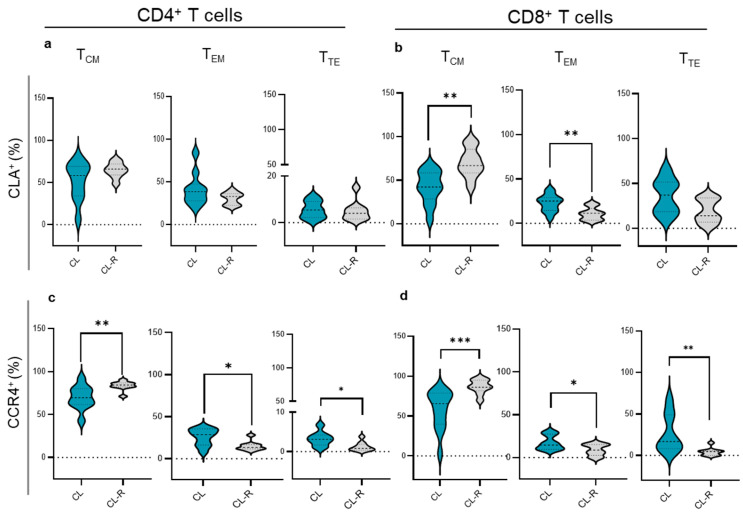
Memory T cell subsets after recovery of CL patients. (CL) Cutaneous leishmaniasis patients (blue) and (CL-R) CL recovered patients (light gray). Memory T cell populations expressing CLA or CCR4 were determined as central memory (T_CM_, CD45RO^+^CD27^+^), effector memory (T_EM_, CD45RO^+^CD27^—^), and terminal effector (T_TE_, CD45RO^—^CD27^—^) CD4^+^ T cells (**a**–**c**) and CD8^+^ T cells (**b**,**d**). Significant differences between active disease and recovery were assessed using the Mann–Whitney test. (*) *p* < 0.05; (**) *p* < 0.01; (***) *p* < 0.001.

The frequencies of memory T cell subsets expressing chemokine receptors were further analyzed according to the clinical outcome. We found higher frequencies of T_CM_ and decreased T_EM_ CLA^+^CD8^+^ T cells in CL recovered patients compared with those with active CL infections (Figure 4b). Similarly, CL recovered patients displayed increased T_CM_ and diminished T_EM_ and T_TE_ CD4^+^CCR4^+^ and CD8^+^CCR4^+^ (Figure 4c,d) cells compared with patients with skin ulcers (Figure 4d). No variations were detected when analyzing CCR6, CCR10, and CCR3 memory populations.

### 3.4. Plasma Concentrations of CCL17 and CCL20 Chemokines

**Figure 5:**
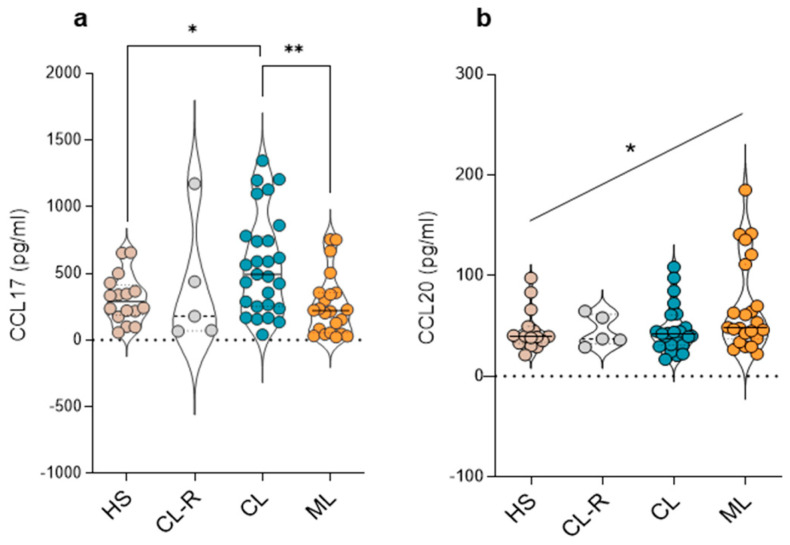
Plasma levels of skin and mucosal homing chemokines. (**a**) CCL17 and (**b**) CCL20 chemokine plasma concentration. (HS) Healthy subjects (gray), (CL-R) cutaneous leishmaniasis recovered patients (light gray), (CL) cutaneous leishmaniasis patients (blue), and (ML) mucosal leishmaniasis patients (orange). Differences between groups were recorded using the Kruskal–Wallis test and Dunn’s post- test. (*) *p* < 0.05; (**) *p* < 0.01. The asterisk over the diagonal line indicates a positive trend using a one-way ANOVA with a post-test for linear trends (*p* < 0.05).

Next, we determined the plasma amounts of CCL17 and CCL20. CCL17 in combination with CCR4 mediates homing to cutaneous sites of specific lymphocyte subsets while CCL20 is a chemoattractant for CCR6-expressing cells. Higher plasma CCL17 levels were found in CL patients compared with ML patients and healthy subjects (Figure 5a). In contrast, a positive trend toward higher CCL20 levels was observed in ML patients (Figure 5b). CL recovered cases showed similar concentrations of CCL17 and CCL20 when compared to the active CL group (Figure 5a,b).

### 3.5. The Duration of the Disease and Its Relation with Plasma Chemokine Concentrations Targeting Skin and Nasopharyngeal Mucosa

**Figure 6:**
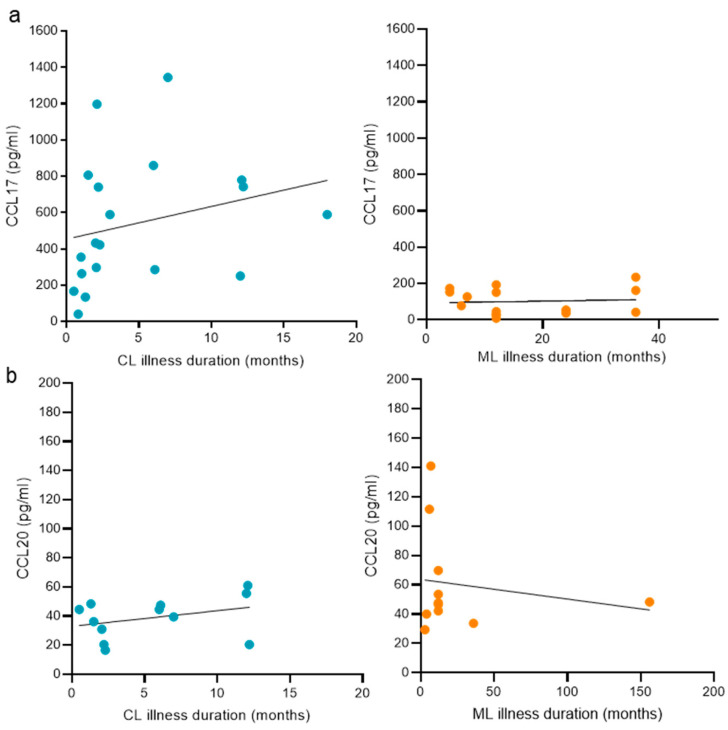
Disease duration and plasma concentration of CCL17 and CCL20 in TL. Linear correlation analysis between disease duration in patients with cutaneous leishmaniasis (CL) or mucosal leishmaniasis (ML) and (**a**) CCL17 and (**b**) CCL20. Healthy subjects (HS), patients with cutaneous leishmaniasis (CL), and patients with mucosal leishmaniasis (ML). Simple linear regression analysis: (**a**) CL, R² = 0.0659; ML, R² = 0.0718. (**b**) CL, R² = 0.1151; ML, R² = 0.0289.

We aimed to analyze the relation between disease duration and CCL17 or CCL20 levels in patients with CL, where cases were comprised within 0.5 months from the appearance of the first skin lesion, and in patients with ML, with samples showing a disease duration of up to 156 months from the onset of the first mucosal lesion. Among the study groups analyzed, we found no association between CCL17 (Figure 6a) or CCL20 (Figure 6b) and the duration of the disease.

### 3.6. CCL17 and CCL20 Production in Culture Supernatants and Cytokine Modulation in Response to Leishmania Antigens

**Figure 7:**
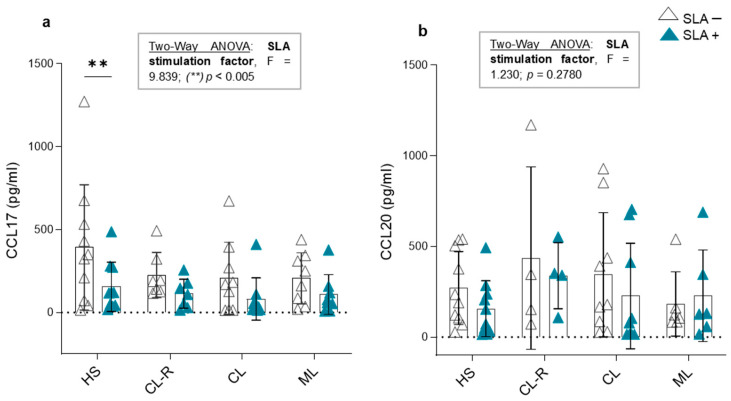
In vitro production of CCL17 and CCL20 in the presence or absence of *Leishmania* sp. antigens. Cytokine levels in culture supernatants of (**a**) CCL17 and (**b**) CCL20 in the presence (blue triangles) or absence (white triangles) of soluble *Leishmania* sp. antigens (SLAs) from peripheral blood mononuclear cells (PBMCs) of healthy subjects (HS), recovered cutaneous leishmaniasis patients (CL-R), cutaneous leishmaniasis patients (CL), and mucosal leishmaniasis patients (ML). Two-way ANOVA with Bonferroni’s post-test.

Using in vitro cultures, we evaluated whether SLAs have a direct effect on the production of CCL17 and CCL20 by PBMCs in patients with active CL, CL after achieving clinical recovery, or ML and healthy subjects. To analyze this aspect of the study, we applied a two-way ANOVA statistical test, as it allows us to determine how a response variable (CCL17 and CCL20 production) is affected by two factors (clinical presentation and SLA stimulation). The results of this test are shown in Figure 7.

Regarding CCL17 concentration in the supernatant, we determined that stimulation with *Leishmania* antigens had a significant inhibitory effect on CCL17 production, regardless of clinical presentation or disease outcome (two-way ANOVA: F = 9.839, *p* < 0.005; Figure 7a). This effect was particularly observed in the healthy subject group, where CCL17 levels significantly decreased in the presence of SLAs (Figure 7a). In contrast, SLA stimulation had no effect on CCL20 production by PBMCs in any of the studied groups (two-way ANOVA: F= 1.230, *p* = 0.2780; Figure 7b). These results support that soluble proteins of *Leishmania* spp. might modulate CCL17 expression independently of previous contact with the parasite.

**Figure 8:**
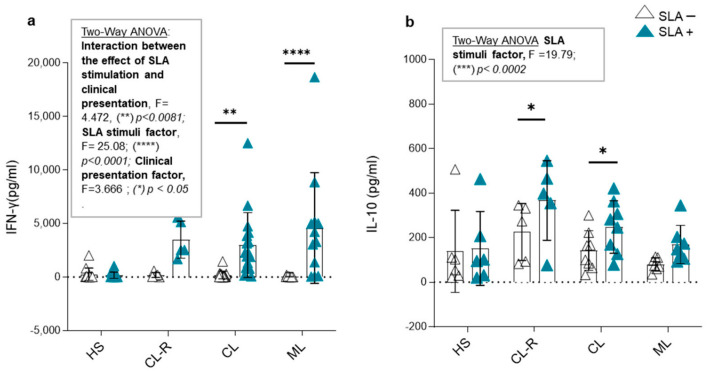
In vitro production of IFN-γ and IL-10 in the presence or absence of *Leishmania* sp. antigens. Cytokine levels in culture supernatants of (**a**) IFN-γ and (**b**) IL-10 in the presence (blue triangles) or absence (white triangles) of soluble *Leishmania* sp. antigens (SLAs) from peripheral blood mononuclear cells (PBMCs) of healthy subjects (HS), recovered cutaneous leishmaniasis patients (CL-R), cutaneous leishmaniasis patients (CL), and mucosal leishmaniasis patients (ML). Two-way ANOVA with Bonferroni’s post-test.

Subsequently, we measured the levels of the pro-inflammatory cytokine IFN-γ and the regulatory cytokine IL-10 in the culture supernatants of PBMCs from the studied groups. The results were analyzed using the two-way ANOVA statistical test. When examining IFN-γ concentration, we identified a significant interaction between the effect of SLA stimulation and clinical presentation, with markedly elevated cytokine levels in ML patients, followed by CL patients (two-way ANOVA: F = 4.472, (**) *p* < 0.0081; Figure 8a), while levels remained at baseline in healthy subjects.

On the other hand, SLA stimulation induced IL-10 production (two-way ANOVA: F = 19.79, (***) *p* < 0.0002; Figure 8b), but its interaction with clinical presentation was not significant. However, only patients with active CL and those who achieved recovery from their skin lesions (CL-R) showed a significant increase in IL-10 production ((*) *p* < 0.05; Figure 8b). Regarding the cytokine environment produced in response to the parasite, PBMCs from patients with cutaneous forms produced a compensatory regulatory effect in the presence of *Leishmania* sp. antigens. This balance between pro-inflammatory and regulatory cytokines was not observed in patients with mucosal involvement.

## 4. Discussion

Tegumentary leishmaniasis presents a broad spectrum of manifestations, encompassing single or multiple cutaneous ulcers and nasopharyngeal lesions [18]. Mucosal lesions usually appear months or years after the primary skin lesion, as a possible consequence of the antigen persistence in the host. The failure of complete elimination of the parasite is well documented by the findings of *Leishmania* sp. amastigotes within cutaneous scars [18,19] or by the reactivation of the infection often observed after immunosuppressive therapies [20]. In this context, the host needs to adapt their immune responses to the location selected by the parasite through the different stages of the disease. Hence, the secreted chemokines and the T lymphocytes recruited to the affected sites play a major role in defining the immune mechanisms that control the infection. The present work demonstrated a modulation of chemokine receptors on total T cells only in the presence of mucosal involvement, with an upregulation of CLA, CCR4, CCR3, and CCR6 on CD4^+^ T cells and CCR3, CCR6, and CCR10 on CD8^+^ T cells. The increased expression of a targeted panel of chemokine receptors involved in skin and mucosal tropism in ML might reflect the expansion of the parasite to nasal mucosa, mobilizing more T cell subsets. In accordance with this assertion, when analyzing memory T cell populations, the changes in CL patients were restricted to subsets expressing CLA and CCR4, while ML patients demonstrated alterations in memory T cells expressing CLA, CCR4, CCR6, and CCR10. The expression of CLA and CCR4 has been associated with the pathogenesis of several skin disorders including autoimmune and oncological diseases [21,22,23,24,25]. Meanwhile, CCR10 and CCR6 play a role in homeostasis mechanisms during skin healing processes [26,27,28] and mucosal inflammation [29,30,31,32,33,34,35], respectively. The results of the upregulation of cutaneous chemokine markers on total and memory T cell subpopulations are indicative of possible persistence of the parasite in cutaneous sites during mucosal disease. In addition, the increase in effector memory/terminal effector T cells at expenses of a decrease in central memory T cells expressing chemokine receptors found here in tegumentary leishmaniasis reinforces previous findings of the chronic activation of T cells with effector functions mainly observed during mucosal cases [36]. Herein, we have provided information of the homing direction of these peripheral memory T cell populations, allowing us to recognize the profile of T cells infiltrating lesions, which play a central role in lesion injury.

Previous reports by us and others demonstrate that the increase in effector memory T cells is accompanied by signs of T cell exhaustion in total CD8^+^ T cells, with a late differentiated (CD27^—^ CD28^—^phenotype, increased expression of senescence markers, and cytotoxic molecules [4,37]. The present work demonstrates that CD4^+^ T cells represent the main subset implicated in the modulation of the memory profile expressing homing markers in CL, while changes in these markers in CD8^+^ T cells were only observed in ML, in correspondence with the exacerbation of cytotoxic functions that characterize this clinical form [38,39,40]. The present results demonstrating the predominance of effector T cells expressing chemokine receptors directed to skin and mucosal tissues could be used in future experimental studies to test the selective blocking of chemokine receptors aiming to diminish possible pathogenic effects of T cells. In this sense, Sacramento et al. [41] recently demonstrated the therapeutic potential of blocking CCR5, leading to a decrease in CD8^+^ T cell recruitment to leishmanial lesions in mice infected with *L. (V.) braziliensis*, ameliorating the destructive inflammation mediated by these lymphocytes.

The implication of CLA and CCR4 described here during active tegumentary leishmaniasis is in accordance with their increased expression previously reported in cutaneous lesions [10,42] and nasal mucosa samples [11]. Moreover, the significant decrease in the frequencies of effector memory T cells together with an increase in central memory T cells expressing CLA and CCR4 after the healing of cutaneous lesions is consequent with the decrease in the parasitic load achieved after a good response to treatment. It is known that central memory T cells have a high proliferative capacity after a secondary stimulus and constitute the main reservoir of effector memory T cells for future encounters with the antigen [43]. ML recovery could not be evaluated in this work, because the majority of the patients showed poor response and relapses after different therapeutic regimens. However, modulation of the chemokine receptors after CL recovery supports their possible usefulness as markers to corroborate the success of the treatment. In practice, patients diagnosed with tegumentary leishmaniasis receive a medical recommendation of periodical follow up [44]. The possible increase in effector memory T cells expressing CLA and CCR4 could alert us about cutaneous reactivation or the appearance of higher numbers of effector memory T cells expressing other receptors as CCR6 could aid in predicting the risk of lesion dissemination to mucosal sites. A progressive increase in the expression of these receptors over time may serve as a reliable indicator of disease progression, potentially enabling earlier clinical decision-making before further deterioration occurs.

Regarding CLA^+^ central memory T cells, while active CL patients showed a co-expression profile of CCR4 and CCR6 similar to that in healthy subjects, ML patients demonstrated a predominance of central memory CCR4^−^CCR6^−^ CLA^+^CD4^+^ T cells, which is associated with Th1 CD4^+^ T cells [45]. This profile might be involved in the exacerbation of inflammation, already described in the context of ML, but functional studies, currently being conducted in our laboratory, are needed to confirm this observation.

Additionally, we showed higher levels of CCL17, the ligand of CCR4, in CL patients compared with ML patients. The elevation of CCL17 has been associated with the progression of skin inflammatory diseases such as atopic dermatitis [46] and also reported in patients with post kala azar dermal leishmaniasis [47]. The findings presented here suggest that in tegumentary leishmaniasis, CCL17 is elevated in the presence of dermal lesions, but this elevation is not sustained in plasma when mucosal involvement appears. Meanwhile, the opposite situation occurs when analyzing CCL20, the ligand for CCR6, with a modest increase in ML cases. CCL20 has been associated with disease severity and mucosal inflammation in gastrointestinal diseases and autoimmune disorders [48,49,50]. Thus, CCL17 and CCL20 showed distinct modulation depending on the clinical form of tegumentary leishmaniasis.

Chemokine production serves to facilitate a protective immune response of the host; however, many infections, including those caused by *Plasmodium* spp., *Leishmania* spp., and *Toxoplasma gondii*, can assist their spread throughout the body by modulating chemokine production, leading to leukocyte infiltration to the site of infection [51,52]. Our in vitro assays showed that *Leishmania* spp. inhibit CCL17 production by PBMCs in CL patients, ML patients, and healthy subjects, but do not alter CCL20 release. Suppression of CCL17 could disrupt Th2-associated immune responses, leading to a shift toward a pro-inflammatory environment which favors the arrival of monocytes able to take up parasites, allowing for their dissemination. According to this explanation, the influx of inflammatory cells was already reported in the in vitro interaction of *L. infantum* with keratinocytes [53]. Another possible mechanism of *Leishmania* spp. could be to alter certain chemokine/chemokine receptor migration pathways. In this sense, Pinheiro et al. [9] demonstrated a decrease in CCR4, the CCL17 receptor, after in vitro *Leishmania* spp. infection, leading to the reduced adhesion of monocytes to connective tissues and suggesting the involvement of this pathway in the migration of these cells from *Leishmania* infection sites. In addition, IFN-γ production in *Leishmania* spp. infection is linked to a protective response and has strong immunomodulatory effects. In murine models, high IFN-γ levels indicate resistance, while IL-10 suggests susceptibility [1,7,54,55]. In humans, this dichotomy is less clear, as the immune response depends on the host’s immune status and the *Leishmania* species involved [55,56,57]. Our study confirms that *Leishmania* sp. antigens strongly modulate IFN-γ production, especially in patients with ML [58]. This increase in IFN-γ suggests persistent activation of CD4^+^ and CD8^+^ T cells. Additionally, the reported results indicate that immune cells are able to maintain their activation capacity even after prolonged infections, as PBMCs from patients with chronic ML demonstrated high antigen-specific inflammatory responses. These findings also support the notion of the pathogenic contribution of pro-inflammatory responses in ML, supporting the deregulated immune activation previously reported [4,56]. Interestingly, both CL and CL-R patients counteracted this pro-inflammatory response through IL-10 production, highlighting the role of regulatory cytokines as a needed balance for lesion resolution and as potential therapeutic strategies for preventing disease progression to chronic mucosal forms [59].

## 5. Conclusions

Altogether, the present work demonstrates the usefulness of a focused panel of chemokine/chemokine receptors to evaluate cutaneous or mucosal compromise and disease outcomes in tegumentary leishmaniasis. Despite the limited set assayed, these chemokine markers enabled us to obtain clear and valuable results contributing to broadening and strengthening the set of receptors yet described in the literature. In addition, our results emphasize the possible pathogenic role of a sustained effector response in chronic TL cases and provide new insights for experimental studies in an attempt to ameliorate the severity of the disease.

## Figures and Tables

**Table 1:** Demographic and clinical characteristics of tegumentary leishmaniasis patients.

Study Groups	N	Age (Years)	Male/ Female	Subjects with Detectable Anti-*T. cruzi* Antibodies (%)	Cutaneous/Mucosal Illness Duration (months)	Time of Complete Recovery (months)
Cutaneous leishmaniasis (CL)	32	40 [12–72]	26/6	9.3	2 [0.5–18]	NA
Cutaneous leishmaniasis recovered (CL-R)	13	42 [19–72]	9/4	6.25	NA	31 [2–72]
Mucosal leishmaniasis (LM)	24	57 [27–86]	17/7	41.66	36 [12–156]	NA
Healthy subjects (HS)	17	37 [29–61]	10/7	0	NA	NA

Results of age, illness duration, and time of recovery are expressed as median [range]. NA, not applicable.

**Table 2:** Expression of chemokine receptors on CD4^+^ and CD8^+^ T cells in patients with cutaneous leishmaniasis and mucosal leishmaniasis.

Cell Marker	CD4^+^ T Cells (%)			CD8^+^ T Cells (%)		
	HS	CL	ML	HS	CL	ML
CLA	10.30 [7.70–12.80]	10.30 [6.70–28.80]	14.80 [7.30–32.80] *	4.65 [2.00–8.70]	3.80 [1.30–13.40]	8.70 [1.30–11.60]
CCR4	14.50 [8.00–30.50]	13.30 [4.30–24.80]	18.40 [7.50–49.30] **	2.60 [1.50–6.00]	3.10 [0.80–6.50]	3.40 [1.70–9.00]
CCR6	6.70 [2.70–9.90]	2.40 [0.50–11.80]	8.40 [0.40–19.70] ≠	1.90 [0.70–7.70]	1.70 [0.70–3.40]	3.30 [0.50–9.60] ≠
CCR3	0.90 [0.50–3.10]	1.45 [0.50–3.80]	2.10 [0.80–4.10] €	0.70 [0.30–2.90]	1.40 [0.40–3.80]	2.60 [1.20–3.10] €
CCR10	6.15 [4.60–9.70]	6.30 [3.20–17.30]	8.10 [4.60–13.30]	2.40 [1.50–4.40]	2.50 [1.50–6.40]	3.75 [1.60–7.70] **

Percentages of expression of CLA, CCR4, CCR6, CCR3, and CCR10 on CD4^+^ and CD8^+^ T cells from HS, healthy subjects; CL, cutaneous leishmaniasis patients; ML, mucosal leishmaniasis patients. Significant differences among groups were evaluated using the Kruskal–Wallis test with Dunn’s post-test (*p* < 0.05: (*) ML vs. HS; (€) ML vs. HS and CL; and (≠) ML vs. CL) and a one-way ANOVA with a post-test for linear trends, *p* < 0.05. (**) A positive trend toward higher expression for ML vs. HS and CL. The results are expressed as the median [range].

## Data Availability

The raw data supporting the conclusions of this article will be made available by the authors on request.

## Abbreviations

The following abbreviations are used in this manuscript:

TL	Tegumentary leishmaniasis
CL	Active cutaneous leishmaniasis patients
ML	Mucosal leishmaniasis patients
CL-R	Recovered cutaneous leishmaniasis patients
HS	Healthy subjects
PBMCs	Peripheral blood mononuclear cells
SLA	Soluble *Leishmania* antigens
T_CM_	Central memory T cells
T_EM_	Effector memory T cells
T_TE_	Terminal effector T cells
